# Prevalence and Psychosocial Predictors of Homophobic Victimization among Adolescents

**DOI:** 10.3390/ijerph16071243

**Published:** 2019-04-08

**Authors:** Antonio J. Rodríguez-Hidalgo, Almudena Hurtado-Mellado

**Affiliations:** Department of Psychology, Cátedra de Cooperación al Desarrollo, Universidad de Córdoba, 14071 Córdoba, Spain; m22humea@uco.es

**Keywords:** homophobic bullying, discrimination, homophobia, cyberbullying, LGBT, sexual orientation, self-esteem, empathy, social skills

## Abstract

Bullying and discrimination seriously damage the development and health of adolescents with non-heteronormative sexual orientation. Adolescents from sexual minorities are more likely to be the object of bullying. This research aims to know more about the prevalence, frequency, and some associated factors and predictors of homophobic victimization in adolescents, depending on their sexual orientation. A total of 820 Secondary Schools students took part in this study (average age = 14.87; SD = 1.72; 48.3% were boys and 51.7% were girls) by filling in a self-report questionnaire. The results showed that adolescents suffer homophobic victimization, regardless of their sexual orientation; however, homosexuals and bisexuals suffered it more frequently than heterosexuals. Homophobic victimization could be associated—in heterosexuals and people with doubts about their sexual orientation, positively with bullying victimization, bullying aggression and cyberbullying aggression. Homophobic victimization could be predicted—in heterosexuals, positively due to self-depreciation, and negatively due to communication and relationship skills; and in homosexuals and bisexuals, positively, because of affective empathy. The results are discussed and new lines of study and intervention are proposed.

## 1. Introduction

The school is a setting where minors establish the basis of their interpersonal relationships, beyond the family setting [1]. In the last decades, cyberspace has extended these two interrelated settings. The new generations of the countries with the highest per capita incomes are digital natives [2]. Different forms of aggression and victimization among peers are experienced not only in the physical, but also in the virtual settings. These may damage their health and welfare as well as hinder, hamper or dash their personal and social development [3].

The students that are different for any reason from the students from the majority group—given their origins, religion or perceived or real sexual orientation—are more likely to be the object of peer victimization [4,5,6]. Adolescence is a period when adolescents are fully discovering and exploring their sexual identity and genre [7], a period in which they can suffer a specific victimization due to homophobic bullying [4,8,9]. The repeated homophobic victimization might lead to depression, anxiety, absenteeism, risk of alcohol abuse, school dropouts, and suicide attempts [10,11,12,13,14,15]. One in four victims of homophobic bullying have had suicidal thoughts due to it [10].

The research carried out by Rivers [16] revealed that—(a) very few victims of homophobic aggressions ask for help to their teachers and only one in six admitted the discriminatory nature of the aggressions; and (b) one in four victims felt harassed by some teacher due to his/her sexual orientation. Nearly two decades after, a study had again showed that male and female adolescents reported the rejection that they suffered within their environment because they had revealed their sexual orientation [17]. In this way, victims of homophobic aggressions find themselves in a paradoxical situation. Revealing their sexual orientation, which is essential to get social support [15], becomes a risk factor for the loss of social support. The public recognition of a sexual orientation different from the heterosexual one causes many adolescents to lose the support from their reference adults—family and teachers among others—and from their peers, due to their prejudices [18]. This worsens even more as a result of the existing “law of silence” in bullying [6], which empowers bullies even more and makes victims more vulnerable and defenseless. In the last years, it has been observed that LGBT adolescents seek more social support and online social relationships than those adolescents who are not LGBT [3].

To develop policies, attention, and psychoeducational strategies for the prevention and eradication of homophobic victimization, further knowledge about this phenomenon is needed, as it is related to bullying, cyberbullying, and discrimination [19,20]. The present study is focused on homophobic victimization suffered by male and female adolescents, with the aim of knowing more about their prevalence and about some of the psychosocial predictors, regarding adolescents’ sexual orientation.

### 1.1. Bullying, Cyberbullying, and Discrimination: Approach to the Study of Homophobic Victimization

When a student is harassed in an intentional and repeated way, and under a power imbalance, bullying occurs [21]. This phenomenon includes different types of behaviors, from mockeries and insults to systematic isolation, threats, and physical aggressions [22,23,24]. Many of these behaviors of aggression and victimization are also produced by means of the use of Information and Communication Technologies (hereinafter ICT). When these cyber behaviors are developed repeatedly, intentionally, and under a power imbalance, cyberbullying occurs [25]. More and more studies show that there is certain overlap between bullying and cyberbullying in adolescence [26,27].

Bullying has a prevalence that ranges from 20% to 60% [23,24,28,29]. Physical bullying appears to be less frequent, whilst verbal bullying seems to be the most frequent [23,24,30]. Over time, the bully subdues the victim, more and more, and the victim perceives him/herself more defenseless, vulnerable, and unable to reverse the situation—victimization [31]. This phenomenon has got all sorts of harmful consequences, such as negative implications in the academic performance and physical and mental health problems [24,32,33,34,35]. Some studies have shown that the victim rate of bullying is quite higher in LGBT adolescents than in heterosexuals [3,36,37]. A study carried out in Spain evidences that the LGBT victims that suffered bullying was almost double, in percentage, the number of heterosexual victims [19].

Cyberbullying has a prevalence ranging from 5% to 50% [28,38,39,40]. Cyber victims have a lot of trouble to defend themselves, because with the use of cyberspace, the bully can make their content reach many people, yet the cyber aggressor remains anonymous [25]. Cyber victimization is specially harmful due to the own characteristics of cyberspace—absence of guardians who avoid aggressive actions over minors, a great diffusion capacity of harmful contents, and a low possibility to delete or forget them totally [41]. Several studies show a greater involvement of LGBT adolescents in cyberbullying as victims [3,20], especially in their more serious forms [19].

Homophobic victimization emerges by means of the bullies’ attacks on the LGBT people—lesbian, gay, bisexual, and transgender—and to the people that, despite not being included in this group, are considered as individuals out of the heteronormativity of the group [42,43]. Homophobia is a set of negative ideas about the attitudes out of the heterosexuality schemes that foster discriminatory behaviors [4,44,45]. It has been observed that female and male adolescents admit it as a fact that not following the fixed gender-based norms implies punishment by classmates and even by adult authority figures [10]. Discrimination is an expression of the stigma or social disregard towards people who perceive themselves with certain different characteristics or identities [42], through the action of a distinct or harmful behavior or even the omission of the interpersonal action [46]. Bullying based on stigma is the common area between intimidation and discrimination [47]. Homophobic victimization emerges when intimidating and discriminatory aggressions among peers are homophobic. This victimization, just like the one caused by traditional or personal bullying, emerges through different sorts of aggressions, such as verbal and physical aggressions [48], and social exclusion and isolation [49]. Among these aggressions, the psychological or verbal aggressions are more common in homophobic victimization [8,43]. The most documented aggression is the homophobic insult [50].

Homophobic bullying has a prevalence ranging from 22% to 87%, which is higher than the prevalence of traditional bullying and cyberbullying [8,42,51,52,53]. Several studies have revealed that the age period, ranging between 10 and 15 years, is the most critical, when homophobic bullying reaches its highest level [7,54,55].

### 1.2. Homophobic Victimization, Self-Esteem, Empathy, and Social Skills

Self-esteem is lower in adolescents that suffer homophobic victimization [8,56]. Self-esteem moderates the relationship between discrimination and its negative results on health [57]. Several studies have outlined self-esteem as a protector factor in bullying and discrimination against the LGBT group. For example, a higher level of self-esteem helps to reduce the negative effects of bullying, based on sexual orientation [15]. The increase of self-esteem is considered as a decrease of the positive association between discrimination and negative affectivity [58].

High empathy is associated with lower levels of homophobia [59]. Some studies have revealed that there is an inverse relationship between empathy and homophobic aggression in adolescents [60]. Nevertheless, no study references have been found about the potential relationship between empathy and homophobic victimization. The scientific literature about personal bullying victimization shows that this is negatively correlated to cognitive and affective empathy [61,62].

Several studies on social skills and personal bullying have found that the victims have a low development of social skills [63]; bullies also have low levels of these skills [64]; and bullies make a bad use of social skills abusing their popularity and leadership to manipulate and harass their peers [65]. In the Vera et al. study [66], social skills were the skills related more closely to bullying, among all other variables that were taken into account. On the other hand, the results of the research carried out by Rodríguez & Noé [67] related assertiveness to a greater ability to overcome bullying victimization situations. It has also been found that victims have; more trouble when coping, a low assertiveness and limited conflict resolution skills [63]. As it can be seen, there is certain scientific knowledge about the relationships between social skills and bullying. However, there is the need of carrying out studies on the relationship between homophobic bullying and social skills, such as communication and relationship skills, assertiveness, and conflict resolution skills.

### 1.3. The Present Study

After reviewing the scientific literature, we need to look into the study of homophobic victimization and the potential related psychosocial factors. The objectives of the study are:

1. To know the percentage of adolescents homophobic victims, and the frequency with which adolescents suffer this abuse.
2. To know if homophobic victimization is related to cyberbullying, bullying, self-esteem, empathy, social skills, and age.
3. To know to what extent self-esteem, empathy, and social skills might be predictors of homophobic victimization.
4. To know the possible differences in homophobic victimization between several groups of adolescents, due to their sexual orientation.

Taking into account the state of the art, these were the initial hypotheses:

1. The involvement as a homophobic victim will be higher in homosexual and bisexual adolescents, as well as in those with doubts about their sexual orientation, as compared to heterosexual adolescents.
2. The frequency of homophobic victimization will be higher in homosexual and bisexual adolescents, as well as in those with doubts about their sexual orientation, as compared to heterosexual adolescents.
3. Self-esteem will be a negative predictor of homophobic victimization, regardless of the sexual orientation of adolescents.
4. Empathy will be a negative predictor of homophobic victimization, regardless of the sexual orientation of adolescents.
5. Social skills will be negative predictors of homophobic victimization, regardless of the sexual orientation of adolescents.

## 2. Materials and Methods

### 2.1. Participants

The study comprises a population composed of students from the first year of Secondary Education to the last year of Bachillerato, in public educational centers of the province of Córdoba, Spain. By means of a non-probabilistic sampling technique by accessibility, a sample of 820 participants was obtained (48.3% were boys and 51.7% were girls), whose ages ranged from 11 to 19 years (average age = 14.87; SD = 1.72). Most of the participants considered themselves as heterosexuals (82.2%, n = 674). Additionally, 6% (n = 49) considered themselves as homosexuals or bisexuals and 11.8% (n = 97) declared themselves to have doubts about their sexual orientation.

### 2.2. Procedure

The research had a retrospective, ex post facto, and cross-sectional design. We contacted 9 educational centers to ask them to participate voluntarily in the study. After 5 of these educational centers confirmed their approval, we asked for the authorization of fathers, mothers, or guardians of students. A total of 854 parents/student guardians were contacted, of whom 5 refused to allow their children to participate in the study. Once the consents were obtained, we briefly explained the study to the remaining 849 students. They were informed that the participation was voluntary and anonymous and that the data provided would be processed confidentially; nonetheless, 3 of them refused to participate. After that, we proceeded to deliver the questionnaires on-site. In every group per class, filling those questionnaires took about 30–50 minutes—depending on the age of the group—during the school hours. Of the total of 846 participants, 11 did not adequately complete one or more of the scales used in the study and 15 left some of the items blank. All these participants were discarded for the investigation. This procedure was carried out from December 2017 to May 2018.

The research respected the ethical principles of the Declaration of Helsinki of the World Medical Association and the national laws that regulate the research and the psychologist’s profession. Procedure was approved by the Ethics Committee of the University of Córdoba (PSI2016-74871-R).

### 2.3. Instruments

We used a set of self-reports composed of several questionnaires. The first part was dedicated to collect sociodemographic data, including age, sex, and the academic year. The second part was composed of the following questionnaires.

Sexual Orientation: We followed the model developed by Poteat, Espelage, and Koenig [68] to report sexual orientation by means of the question: “Do you ever feel confused about whether you are homosexual or bisexual?”. Participants had to choose only one answer from the different options—(1) “Never confused because I consider myself to be heterosexual”; (2) “Rarely confused”; (3) “Sometimes confused”; (4) “A lot confused”; or (5) “Never confused because I consider myself to be homosexual or bisexual”.

Homophobic Bullying: We used the Spanish version [27] of an adaptation of the European Bullying Intervention Project Questionnaire—EBIP-Q [69]. The adaptation involved changing the brief introductory explanation that guides the participants when filling the questionnaire. In the original questionnaire, students were guided to answer about bullying situations that they might have experienced, but without indicating the reason. This introduction was changed by another one in order to guide the participants to only answer to homophobic bullying situations: “Now we will ask you about your possible discriminatory experiences within your environment (school, friends, acquaintances) between heterosexuals, homosexuals or bisexuals, due to differences in the sexual orientation or the gender identity of each of them, in the last two months”. Later, they were asked regarding the 14 items of the original tool, 7 items measuring victimization (Factor 1, α = 0.852) (e.g., “Someone has hit me, kicked me or pushed me”) and 7 items measuring aggression (Factor 2, α = 0.774) (e.g., “I have threatened someone”). These questions were measured with a 5-point Likert scale, being 1 “No” and 5, “Yes, more than once a week”. The CFA was carried out where the bifactorial solution of the questionnaire was subjected to contrast. The indices obtained showed a good fit to the observed data (χ^2^ = 330.369, df = 76, CFI = 0.982, IFI = 0.982, NFI = 0.977, NNFI = 0.979, RMSEA = 0.064). The same two dimensions that the original scale of traditional bullying presents were observed.

Self-esteem: The Rosenberg Self-Esteem Scale—RSES—[70] was used, adapted, and validated by Martín-Albo, Núñez, Navarro, and Grijalvo [71]. This scale is composed of ten items, which are divided into two dimensions [72]—five items (e.g., “I am able to do things as well as most people”) on self-confidence (α = 0.772); and five items (e.g., “I feel that I do not have many things to feel proud of”) on self-deprecation (α = 0.821). Each item was answered by means of a point scale, ranging from 1 (“Totally disagree”) to 4 (“Totally agree”).

Empathy: The Basic Empathy Scale—BES [73] was used in a reduced version adapted to Spanish [74]. This scale is divided into two dimensions—four items (e.g., “After being with a friend who is sad for some reason, I often feel sad”), on affective empathy (α = 0.755); and five items (e.g., “I usually realize fast when a friend is angry”), on cognitive empathy (α = 0.745). Each item was answered by means of a point-scale, ranging from 1 (“Totally disagree”) to 5 (“Totally agree”).

Social Skills: The Social Skills Rating Scale [74] was used. It is composed of twelve items, classified in three dimensions—four items (e.g., “When two friends have argued, they usually ask me for help”) on conflict resolution skills (α = 0.666); three items (e.g., “I usually praise my classmates when they do something right”) on assertiveness (α = 0.532); and five items (e.g., “I have difficulty starting a conversation with someone I do not know”) on communication or relationship skills (α = 0.744). Each item was answered by means of a point-scale ranging from 1 (“Absolutely false”) to 7 (“Absolutely true”).

## 3. Results

### 3.1. Prevalence and Frequency of Homophobic Victimization Depending on the Sexual Orientation

In the study sample (N = 820), 23% of participants stated to be homophobic victims. They were divided into groups, depending on their sexual orientation; they declared having been victims of homophobic aggressions: 23.9% were heterosexuals, 19.6% were those who were doubtful about their sexual orientation, and 14.3% were homosexuals or bisexuals. Among these groups, the differences in percentages were not meaningful (χ^2^ = 3.03, *p* = 0.0220).

Several variance analyses were carried out to know if there were differences in the levels of frequency of being the object of homophobic victimization and for each of the ways of homophobic victimization, depending on the adolescents’ sexual orientation—Heterosexual, Doubts about his/her sexual orientation, Homosexual or Bisexual. The Levene’s test was used to know the homogeneity of the variance. In the cases in which Levene’s statistic offered a level of significance lower than 0.05, the Welch’s statistic was used as a reference. When variances were different, the Games-Howell’s test was used to observe the between groups differences. We used the Turkey’s test when variances were equal.

A one-factor ANOVA was used to contrast the average levels of frequency of homophobic victimization, depending on the three groups of sexual orientation. Since the Levene’s statistic offered a significance level below 0.05, we used the Welch’s statistic as a reference. Significant differences were seen, but the effect size was too low F (2, 93) = 7.49, *p* = 0.001, η^2^ = 0.028. The Games-Howell post-hoc comparison for the three groups indicated that homosexuals and bisexuals (M = 9.16, SD = 3.20, 95% CI [8.24,10.08]) showed significantly higher levels of frequency of homophobic victimization than heterosexuals (M = 7.64, SD = 2.32, 95% CI [7.46,7.81]).

A one-factor ANOVA was used to contrast the average levels of frequency for one in seven types of homophobic victimization, depending on the three groups of sexual orientation. In those types where significant differences were detected, the Levene’s statistic offered a significance level below 0.05, so we used the Welch’s statistic as a reference. Only in three of the different types of homophobic victimization, significant differences were detected among some groups, presenting very low effect sizes: “Someone has insulted me” F (2, 91.96) = 7.61, *p* = 0.001, η^2^ = 0.035; “Someone has used offensive words about me with other people” F (2, 91.51) = 5.77, *p* = 0.004, η^2^ = 0.025; and “Someone has spread rumors about me” F (2, 89.31) = 7.59, *p* = 0.001, η^2^ = 0.042. For each of these three types of homophobic victimization, we used the Games–Howell post-hoc comparisons for the three groups of sexual orientation. Homosexuals or bisexuals received more insults (M = 1.67, SD = 1.03, 95% CI [1.38,1.97]) than heterosexuals (M = 1.17, SD = 0.62, 95% CI [1.12,1.21]). Offensive words about the victim spread by other classmates were suffered more by homosexuals or bisexuals (M = 1.47, SD = 0.87, 95% CI [1.22,1.72]) than by heterosexuals (M = 1.13, SD = 0.55, 95% CI [1.09,1.17]). Rumors through third parties were suffered more by homosexuals or bisexuals (M = 1.55, SD = 1.04, 95% CI [1.25,1.85]) than heterosexuals (M = 1.12, SD = 0.51, 95% CI [1.08,1.16]), and more by the ones in doubt about their sexual orientation (M = 1.40, SD = 1.00, 95% CI [1.20,1.60]) than heterosexuals (M = 1.12, SD = 0.51, 95% CI [1.08,1.16]).

### 3.2. Relationship between Homophobic Victimization and Cyberbullying, Bullying, Self-Esteem, Empathy, Social Skills and Age

Pearson bivariate correlations analyses were performed for each of the three groups of adolescents, according to their sexual orientation. These analyses were made to study the possible relationships between homophobic victimization and—cyber victimization, cyber aggression, bullying victimization, bullying aggression, self-confidence, self-deprecation, affective empathy, cognitive empathy, assertiveness, communication/relationship skills, conflict resolution skills, and age (See Table 1). In heterosexuals, homophobic victimization correlated positively with cyber victimization (r = 0.26, *p* < 0.01), cyber aggression (r = 0.20, *p* < 0.01), bullying victimization (r = 0.25, *p* < 0.01), bullying aggression (r = 0.18, *p* < 0.01), and self-deprecation (r = 0.13, *p* < 0.01). In adolescents with doubts about their sexual orientation, homophobic victimization correlated positively with cyber victimization (r = 0.34, *p* < 0.01), cyber aggression (r = 0.33, *p* < 0.01), and bullying victimization (r = 0.47, *p* < 0.01). In homosexuals and bisexuals, homophobic victimization correlated positively with cyber victimization (r = 0.43, *p* < 0.01), bullying victimization (r = 0.60, *p* < 0.01), and affective empathy (r = 0.37, *p* < 0.01).

### 3.3. Predictors of Homophobic Victimization Depending on the Sexual Orientation

A multiple linear regression was used by means of the “Enter” method, in order to observe how the variables considered as explanatory or predictive in the literature, were related to homophobic victimization as a criterion variable (See Table 2). The variables that we considered were the following—self-confidence, self-deprecation, affective empathy, cognitive empathy, assertiveness, communication/relationship skills, and conflict resolution skills. The multiple linear regression technique was used for each of the different groups regarding their sexual orientation—heterosexuals; with doubts about their sexual orientation; or homosexuals or bisexuals. The Durbin–Watson statistic showed values that ranged between 1.80 and 2.01 in the three cases. The collinearity analyses were optimal, with tolerance values between 0.35 and 0.88, with a variance inflation factor (VIF) between 1.21 and 2.90.

For the heterosexual group, 2% of variance in the level of homophobic victimization could be explained by the following variables—self-deprecation (positive predictor), and communication/relationship skills (negative predictor) (See Table 2). For the group of adolescents with doubts about their sexual orientation, none of the variables contemplated, showed a predictive power of homophobic victimization (See Table 2). For the group of homosexuals or bisexuals, 4.7% of variance in the level of homophobic victimization could be explained by the variable of affective empathy (positive predictor) (See Table 2).

## 4. Discussion

The scientific literature shows that the proportion of victims of aggressions by their peers is higher in LGBT adolescents than in heterosexual adolescents [3,19,36,37]. For this reason, it was expected that, in both homosexual and bisexual adolescents, as well as in adolescents with doubts about their sexual orientation, the proportion of victims of homophobic aggressions was higher than the one reported in heterosexual adolescents. However, the results that we obtained do not corroborate this first hypothesis. Among heterosexual adolescents, nearly one in four indicated having suffered homophobic aggressions. This is consistent with the fact that heterosexuals were also the object of homophobic aggressions [43]. Among adolescents with doubts about their sexual orientation, one in five admitted to have suffered homophobic aggressions. Among homosexual or bisexual adolescents, almost one in six showed to have suffered homophobic aggressions. The involvement as a homophobic victim was very similar among the three groups of adolescents regarding their sexual orientation, without significant differences. One in four adolescents—regardless of their sexual orientation—has been victim of homophobic aggressions.

Based on the fact that adolescents with non-heteronormative sexual orientation are more likely to suffer peer victimization [4,6], it was expected that the frequency of homophobic victimization was higher in adolescents with doubts about their sexual orientation and in homosexual and bisexual adolescents, in comparison to heterosexual adolescents. This second hypothesis was partially corroborated as homosexual or bisexual adolescents suffering a higher frequency of homophobic victimization than their heterosexual peers.

Among the seven types of homophobic victimization that we studied, significant frequency differences were reported in three of them, depending on the adolescents’ sexual orientation. Homosexual or bisexual adolescents suffered higher levels of frequency of homophobic insults, homophobic offensive words, and homophobic rumors, than heterosexual adolescents. Those adolescents with doubts about their sexual orientation also showed higher levels of frequency of homophobic rumors than their heterosexual peers. Homosexuals or bisexuals were more likely to be submitted to verbal homophobic aggressions, which is consistent with some previous findings [8,43].

Among adolescents, regardless of their sexual orientation, levels of age, and social skills—assertiveness, communication/relationship skills, and conflict resolution skills—cognitive empathy, and self-confidence, had no relation with homophobic victimization levels. In heterosexuals, higher levels of homophobic victimization show higher levels of cyber victimization, cyber aggression, bullying victimization, bullying aggression, and self-deprecation, and vice versa. Within adolescents with doubts about their sexual orientation, higher levels of homophobic victimization showed higher levels of cyber victimization, cyber aggression, bullying victimization, and vice versa. In adolescents with doubts about their sexual orientation, higher levels of homophobic victimization showed higher levels of cyber victimization, cyber aggression, bullying victimization, and vice versa. For the three groups of adolescents, based on their sexual orientation, the levels of cyber victimization and bullying victimization were positively associated with homophobic victimization levels. In both heterosexual adolescents and adolescents with doubts about their sexual orientation, levels of cyber aggression were positively associated with homophobic victimization levels.

The predictive factors of homophobic victimization changed in presence and in weights, depending on the sexual orientation. For heterosexual adolescents, homophobic victimization could be positively predicted by self-deprecation, and negatively predicted by communication and relationship skills. For those adolescents with doubts about their sexual orientation, homophobic victimization could not be predicted on the basis of self-esteem, empathy, or social skills. For homosexual or bisexual adolescents, homophobic victimization could be predicted positively by affective empathy.

The third hypothesis raised the issue that self-esteem would act as a negative predictor of homophobic victimization. This was only corroborated in the heterosexual group. The lack of the predictive ability of self-esteem, on homophobic victimization in adolescents with doubts about their sexual orientation, and on homosexual or bisexual adolescents, turned away from the conclusions drawn by some previous studies [8,15,56]. The fourth hypothesis, related to the issue that empathy was a negative predictor of homophobic victimization, was only corroborated in homosexuals or bisexuals. The fifth hypothesis was only corroborated for heterosexuals; that social skills—only the communication/relationship skills—acted as negative predictors of homophobic victimization. Therefore, not all our expectations about homophobic victimization, based on previous studies about bullying and empathy [61,62] and social skills [63,67] were met.

## 5. Conclusions

The evidence obtained in the present study enables us to conclude that homophobic victimization is a phenomenon that adolescents suffer, regardless of their sexual orientation, but it is suffered more by homosexual or bisexual adolescents than by heterosexuals. Age does not seem to be related to the suffering of homophobic victimization.

Homophobic victimization seems to be linked to victimization and aggression in the physical space and in cyberspace, both in adolescents with doubts about their sexual orientation and in heterosexuals. However, in homosexual and bisexual adolescents, homophobic victimization is linked to face-to-face and cybernetic victimization, but not to aggression in either of these two levels.

It seems that heterosexual adolescents are more likely to be the object of homophobic aggressions, as they suffer more aggressions and attack others in the physical space and cyberspace; they value themselves more negatively and they self-perceive themselves with low skills of expression and communication. This observation was concordant with the predictive patterns of homophobic victimization in heterosexuals, which is very similar to the predictive patterns of bullying and cyberbullying victimization that have been described in the scientific literature, in which self-esteem and social skills play a relevant role [41,63]. However, the types of associations of violent phenomena detected and the predictive patterns that we found for homophobic victimization, among peers with doubts about their sexual orientation and homosexuals or bisexuals, are not consistent with the findings regarding bullying and cyberbullying. Adolescents with doubts about their sexual orientation seem to be more likely to be the object of homophobic aggressions if they receive more aggressions face-to-face and in cyberspace, and if they emit more cyber-aggressions. However, homosexual and bisexual adolescents are more likely to be the object of homophobic aggressions if they receive more aggressions in the physical space and in the cyberspace and if they have more affective empathy. This is consistent with the reflection that there are more bullying victims [19,36,37] and cyberbullying victims [3,20] among LGBT adolescents, than among heterosexual adolescents.

The homophobic victimization that we have described in this study, suffered by adolescents regardless of their sexual orientation, could be correlated to the homophobia-driven discrimination [4,44,45], and with punishment from peers towards those who do not meet the established gender-based norms [10]. In this study it was found that—(a) adolescents with doubts about their sexual orientation suffered more frequent, indirect verbal homophobic victimization than heterosexuals; and (b) homosexuals or bisexuals suffered more frequently, both direct and indirect verbal homophobic victimization, than heterosexuals. These reflections are consistent with the theoretical perspective, according to which homophobic victimization is produced by means of attacks directed to individuals and their behaviors, which seem to be out of the heteronormativity of the group [42,43]. Nevertheless, if we take into account the wide percentage of heterosexuals who admit to be homophobic victims, as well as the predictive pattern that we observed for homophobic victimization in this group, it is proposed that there are behaviors that do not aim to punish when someone leaves the heteronormativity group. These behaviors aim to harm or subdue the peer, tagging, or slandering him/her, based on a wide-spread social prejudice, in this case, a discriminatory prejudice towards diversity in sexual orientation. These homophobic aggressions would use social prejudice as another means of personal bullying. Even so, we should not overlook the homophobic content of these aggressions, performed in social settings, as they could have an indirect discriminatory effect in boys and girls that do not consider themselves as heterosexuals. This way, homophobic victimization in adolescents would be both discrimination as a social expression of the stigma [42] and the disrespect towards those people who perceive themselves differently, due to their sexual orientation.

Based on the associations detected between personal bullying, cyberbullying, and homophobic victimization, psychoeducational programs of prevention of homophobic discrimination should consider contents and strategies addressing these two phenomena. The programs of prevention of bullying and cyberbullying, should also include contents and strategies directed to homophobic aggressions and victimization. In this programs, they should work with adolescents about how to reduce their negative self-esteem or self-deprecation, without putting aside the possibility of building positive self-esteem, empathy, and social skills, since other studies have shown that they are related to bullying and cyberbullying [8,60,66]. The protocols when there are bullying or cyberbullying cases that are activated in schools should explicitly consider measurements to detect suspected cases of homophobic aggressions and victimization and their handling. It would also be convenient to develop a specific training on risks and safe use of the Internet and social networks, for all students. It is known that LGBT adolescents seek more social support and relationships in the cyberspace than the rest of their classmates [3].

The prevention of homophobic victimization in adolescence must consider an education to eradicate sexist stereotypes and heteronormative genders. These stereotypes create social barriers for the inclusion of adolescents who do not follow the default heteronormativity in the peer group [10,45] and, to a large extent, they are the origin of discriminatory prejudices and behaviors [4,44,75]. Educational measures are needed to standardize the image of adolescents with doubts about their sexual orientation, homosexuals, or bisexuals, among their peers, so that they are not perceived as unequal and, therefore, of a lower status. It would be convenient to develop educational measures so that adolescents with non-heteronormative sexual orientations (a) can be empowered and allowed to be assertive in social equity with the other peers; and (b) can be trained in coping strategies of homophobic aggressions that enable them to leave or overcome the dynamics of bullying and discrimination. Additionally, it should be advisable to train the members of educational communities —not only students—to (a) make them aware of the harm of an abusive behavior and homophobic social exclusion and (b) to teach them to develop preventive and palliative actions for these phenomena. To foster the effectiveness of the educational strategies is crucial that the different members of the school community and educational agents collaborate in schools. The cooperative work among families, students, teachers, with the support of specialized external actors, have shown to be a protector factor in case negative consequences of homophobia emerge in the victims [15,43], and it can be a key factor for its prevention.

The present study has some limitations and opens the door to develop some new lines of research. One of the limitations was the size of the sample, which led to the fact that the subgroups of non-heteronormative sexual orientation had a small size. The type of the methodological design—cross-sectional and with self-reports—has some limitations, although it has been fitted to the aims and initial hypotheses. In further studies, the aim should be a wider sample, carrying out several data collection about the same subjects, and the combination of the use of self-reports and hetero-reports. A longitudinal study will enable to draw conclusions, beyond prediction, regarding the precursor and protector factors of homophobic victimization. The triangulation of self-reports and hetero-reports will allow us to have further data to delve into the interpretation of homophobic victimization as an interpersonal phenomenon. Additionally, in future studies about homophobic victimization, we aim to include other variables regarding the disclosure—or not—of their sexual orientation, since the scientific literature shows different reflections on their benefits or prejudices regarding social support [15,17,18]. This knowledge could be worthy to help victims and to move forward in the prevention of this harmful violent phenomenon among peers. It is a priority to progress in the study of homophobic bullying, for its prevention and eradication. Co-existence, as well as educational and social inclusion of people with different sexual orientations, largely depend on this.

## Figures and Tables

**Table 1:** Matrix of bivariate correlations between the variables by sexual orientation.

Variables	Heterosexual	Doubts About His/Her Sexual Orientation	Homosexual or Bisexual
	Homophobic Victimization		
Cyber victimization	0.26 **	0.34 **	0.43 **
Cyber aggression	0.20 **	0.33 **	0.12
Bullying victimization	0.25 **	0.47 **	0.60 **
Bullying aggression	0.18 **	0.15	−0.19
Self-Confidence	−0.01	−0.10	0.06
Self-Deprecation	0.13 **	0.14	0.05
Affective Empathy	0.01	−0.07	0.37 **
Cognitive Empathy	0.04	−0.61	0.14
Assertiveness	0.01	0.04	0.26
Communication/Relationship Skills	0.01	0.13	−0.03
Conflict Resolution Skills	0.03	−0.11	0.17
Age	−0.02	−0.07	0.12

Note: ** *p* < 0.05.

**Table 2:** Regression models for homophobic victimization by sexual orientation.

Sexual Orientation	Corrected R^2^	Model	Unstandardized Coefficients		Standardized Coefficients	*T*	*P*	95% Confidence Interval for B	
			B	Standard Error	Beta			Lower Bound	Upper Bound
Heterosexual	0.020	(Constant)	5.617	0.898		6.254	0.000	3.854	7.381
		Self-Confidence	0.048	0.039	0.060	1.249	0.212	−0.028	0.124
		Self-Deprecation	0.129	0.031	0.200	4.123	0.000	0.068	0.191
		Affective Empathy	−0.009	0.028	−0.015	−0.331	0.741	−0.065	0.046
		Cognitive Empathy	0.020	0.031	0.031	0.642	0.521	−0.041	0.080
		Assertiveness	0.016	0.031	0.024	0.528	0.598	−0.044	0.077
		Communication/Relationship Skills	−0.028	0.013	−0.087	−2.051	0.041	−0.054	−0.001
		Conflict Resolution Skills	0.000	0.022	0.001	0.019	0.985	−0.043	0.043
Doubts about his/her sexual orientation	0.018	(Constant)	7.456	4.183		1.782	0.078	−0.858	15.769
		Self-Confidence	−0.002	0.169	−0.002	−0.011	0.991	−0.338	0.334
		Self-Deprecation	0.087	0.128	0.107	−675	0.501	−168	0.341
		Affective Empathy	−0.040	0.122	0.039	−0.331	0.742	−0.283	0.202
		Cognitive Empathy	−0.041	0.164	−0.033	−0.252	0.802	−0.367	0.284
		Assertiveness	0.148	0.135	0.139	1.096	0.276	−0.120	0.416
		Communication/Relationship Skills	0.051	0.053	0.106	0.961	0.339	−0.054	0.155
		Conflict Resolution Skills	−0.116	0.107	−0.151	1.079	0.284	−0.329	0.097
Homosexual or Bisexual	0.047	(Constant)	−1.674	5.565		−0.301	0.765	−12.912	9.564
		Self-Confidence	0.250	0.214	0.281	1.172	0.248	−0.181	0.682
		Self-Deprecation	0.148	0.175	0.191	0	0.402	−206	0.502
		Affective Empathy	0.336	0.152	0.404	2.210	0.033	0.029	0.644
		Cognitive Empathy	0.072	0.165	0.083	0.440	0.662	−0.260	0.405
		Assertiveness	0.118	0.139	0.153	0.852	0.399	−0.162	0.398
		Communication/Relationship Skills	0.004	0.072	0.008	0.050	0.960	−0.142	0.149
		Conflict Resolution Skills	−0.141	0.037	−0.230	−1.029	0.310	−0.418	0.136

Gray background: significant variables.
